# Progressive Dentistry

**Published:** 1896-01

**Authors:** J. C. Townsend

**Affiliations:** Colorado Springs, Col.


### PROGRESSIVE DENTISTRY.

BY J. C. TOWNSEND, D.D.S., COLORADO SPRINGS, COL.

PROGRESS in any and every form of education and enterprise is the result of reinforcing precept with experience. Education is the road by which we climb the steep and rugged heights of any pursuit. It is not only the medium by which we struggle for daily bread, but the source of all light, liberty, love, and lasting happiness. Progress is fostered by the momentum of fever and passion of ambition, by the glory of attainment, the mortification of defeat, the attrition of man with man in association and co-operation. Life is thus valuable as progress accompanies our efforts. Wisdom is often gained from failure; sympathy with others, from anguish suffered by ourselves; strength from conquest; courage in adversity, because we see how little fate can really harm us; humility in prosperity, because we learn how accidental and superficial prosperity really is; charity, because we find with what slow progress the greatness and littleness of humanity is distributed; kindness, because the fire we kindle on our brother's hearthstone warms and irradiates our own thoughts, and often submerges our failures into success.

We progress generally by perseverance and union of heart; it is no spontaneous blossom or flowery beds of ease, but reminds us of the breeze that blows through the garden, catching the odors, delightful or repulsive, according to the flowers therein; so out of our lives and into the viewless world beyond we carry nothing but results. Thus our real selves or our talents are the resultants of many diverse forces; and the object of a well-defined specified education is to marshal these forces so as to produce strength, beauty, and wealth out of seeming weakness and discord. In this broad sense, our zeal for success and progress is necessarily co-extensive with our lives. Our progress, however, is limited, without unison of accord, and, of course, is optional. But we should aim at a certain definite work, and its merit is in direct ratio to the value we place on our talents.

In this world every man that wishes to succeed must be a worker and a fighter of some sort; and carefully-selected knowledge, well-earned and learned, is the Vulcan that forges our instruments, sharpens our tools, compounds our medicine, and prescribes all means for a cure.

Well tempered must be the steel and keen the blade, for in the battle of life each man has only what he can seize and hold against unrelenting competition. The brain that thinks and weighs, the eye that questions and discovers, the ear that listens and hears echoes of voices inaudible to disinterested ones, the keen intellect that keeps step with the march of sages, philosophers, and writers of every age and nation, yields a heart not only brave, but full of sympathy for our greatest advancement and success, physically, financially, and spiritually.

The characteristics of our nature must be at least threefold, the fundamental principles of which are useful, ornamental, and elevating.

While recognizing the wide province of a thorough dental education, it is illogical for practical purposes to draw a distinction between the young professional man or his elder changing his location of city or State, as it is necessary for us all to be always fully equipped; but the general principles of honor, truthfulness, justice, charity, courtesy, public spirit, and due regard for the rights of others should prevail, and be urged by every man worthy of his calling.

We have specialized our work; it is theoretical as well as practical, and the underlying principles are illustrated by application.

We are glad to know that our colleges are striving, as well as individual benefactors, to train or teach us how to do, not simply how to know. With a specially adapted course of study, and a thorough, competent instructor and examiner, the object is near and clear, and methods direct and effective. Dentists are biased in favor of the tracks trodden by themselves during previous years. The so-called dentists, of all professional people, are under the greatest temptations to fall into ruts, and to regard with suspicion unfamiliar plans or methods.

We think that new advantages can be gained only by sacrificing older ones, and it is no easy thing to strike a judicial balance between the problematical ounces of profit in one scale and the certain and tried ounces in the other, especially with such stanch conservatives as we try to be. Besides, empiricism is as common in our profession as in medicine, and many of the so-called reforms are half-truths, and half-truths are proverbially more misleading than falsehoods. Experiment shows that lectures and demonstrations are not only beneficial and elevating, but entertaining and healthful on whatever subject, provided they are delivered to seekers of knowledge.

As a profession, we have made great progress in many ways, and yet much, however, remains to be done; and at best we are only standing or encamped on Pisgah, overlooking the land flowing with milk and honey; but to advance too fast is more disastrous than to stand still. Yet, if hurry is ruin, rest is stagnation. The great problem is the bringing us into more immediate relations with things themselves rather than with this absurd method of written examinations and the perusal of long articles.

An ideal is never so real or substantial as a thing. So far as possible the hand should touch, the ears hear, the eyes witness, the feet traverse that which the brain desires to know. So we may realize what we conceived. Our minds are crammed with facts and theories more or less distorted and ill-understood, and useless because unapplied and unrelated to the real experiences of life.

The public should think we have real enthusiasm for learning, and not place us far below the best physician; and, in fact, not below any other profession as to science in our calling. But we believe, while the public is not enlightened, that there is no use of our resting under such condemnation, as all our faculties are throwing out their tentacula for the greatest good and success of our life, as any other profession or people, and our so-called selfishness for gain could be modified by union of thought and action.

Now, the question arises, What should every dentist know, and how can State legislation benefit his life and practice, and raise dentistry to a higher plane? Just what a good dentist in general practice should know is an exceedingly difficult question. Of course, we should have a good academic education; we should be known to have a good moral character, and a diploma from some one of the many reputable dental colleges in this or some other country; should write a thesis on some subject pertaining to dentistry once a year, and deliver the same to a State superintendent, and such thesis should be published in a dental journal or in pamphlet form for general distribution and information.

It is recognized that the chief aim of every man's life is self-support. And closely following comes the desire and natural inclination for the support of others. But equally as important is it that we should be true, not only to ourselves, but to those with whom we deal and are called upon to serve; and last, but not least, comes the desire for fame, and doing good unto our fellow-men. These four elements of a successful and honorable business-man, of whatever calling, constitute the foundation for a good dentist.

Now let us look at the different operations a good dentist is called upon to perform in general practice. The first important duty is to make a proper diagnosis of the conditions, not only of the teeth, but of the mouth and general system. Seldom do we spend enough time on these conditions. But the proper diagnosis is the forerunner of success, and he who has the power to arrive at such conclusions first, or with the most despatch, gets the most out of our profession or any other.

Then comes the adjusting of temperaments, adapting of circumstances, and ability to use the means at hand to remove the cause of all trouble arising from the teeth. Suffice it to say that the dentist is called upon to serve in the capacity of a judge, physician, surgeon, artist, mechanic, teacher, and socialist. He is expected to know the general principles of anatomy, physiology, pathology, chemistry, materia medica, therapeutics, metallurgy, and to be an expert in all operations and mechanism pertaining to dentistry.

But we are not all constituted alike, nor have we the same talents, even when called to the profession of dentistry. And here arises the question, Should every dentist be familiar with all kinds of mechanism and medical treatment in order to practise at all? And should one examination, provided you remain in the same State, suffice without ever raising a voice anywhere again to show that we are alive and doing all in our power, not only to serve the public, but also to elevate our profession?

Where and how can we best show our honest intentions to elevate ourselves and our brother practitioners? First, we would say, by contributing liberally of our income to the support of general education, sustained by a good superintendent; then, through the general association of all practitioners, coming together at least once a year, and not only reading and listening until we become regular drones, but doers as well.

Now, every one can and should contribute something of advantage to the profession, and not only in a pecuniary way, but with the use of his best intellect. What faithful workers we are, so far as it is absolutely necessary, but naturally dilatory when there appears no necessity; but every good-thinking and conscientious man must admit that all that pertains to the general advancement of our profession is a healthy factor in our lives. If so, let us join heart and hand to bring this about, and there will be enough work for all, and to spare.

The question now arises, How can we get the greatest good and sustain the highest standing for our profession? It seems to us that could be accomplished through a State superintendent, elected by our State Society, approved by the governor, and given power of attorney through the Legislature. Now, such a superintendent should act as examiner and instructor for every dentist in the State, and visit every town where there is a dentist, and devote one day every year with each dentist, and give several public clinics and lectures for the dentists and public in all improved methods of practice.

Such a superintendent should be an ideal professional man. His remuneration should be provided for by an assessment of a certain percentage of the professional income of each dentist in the State or district over which he has the supervision. We want to say just here that we think our public school systems set us an excellent example, inasmuch as their teachers have an examination from a State superintendent every year in order to sustain their progress.

We must devote more time to study and research, have our monthly associations wherever there happens to be a half dozen dentists in the same town or city; let each member write a thesis on some subject once at least during the year, and, in reality, take greater pride in our profession, and the public will take greater pride in us, and will reward us accordingly.

The results obtained by having such a superintendent would be that of knowing and practising the best known principles pertaining to dentistry without experimenting so much and so long, and a greatly increased confidence on the part of the public when made aware of such proceedings, and naturally more attention and work for the same; also a greater remuneration for our work, as well as increase of business. So let our motto be, "The greatest means for the greatest good."

We must advance by or through some means at once, and all the time. Let us get down to the foundation and to solid facts concerning our future prosperity, and act on the same at once. Let us get at something on the line of universal progress, and the Great Ruler of all good works will help us and give us the increase.

Now, a man entering our profession should just as well be compelled to keep his knowledge of essential things pertaining to dentistry and add thereto as to be compelled to have in store an abundance when he starts. So let us contribute freely and justly to the support of our best advancement.

A State superintendent should issue a report and give all the information that exists, not only from our State practitioners, but all over the world, and request that each dentist use and try the best methods of operation, and report the results at least every three months. The superintendent should not only enlighten his brother practitioner, but the public as well, and urge regular and thorough attention of the people to their teeth. In other words, he should stir up business and blow the dentist's horn. He could control indolent and worthless practitioners by a public announcement of such in his report to the people of the vicinity in which the intruder lives.

In conclusion, I would say that everything and everybody needs nurture, and dentists especially need association, which should be the best.

Permit me to say that I think the necessary information of all branches pertaining to dentistry should be marked or compiled in book-form by a national board of dental education, and a supplement added from time to time. Now, this means that every dentist who has the greatest pride and success of his profession at heart should be able to learn quickly and thoroughly all the necessary knowledge, and have the same at any time.

